# Case report: Successful treatment of recurrent *E. coli* infection with bacteriophage therapy for patient suffering from chronic bacterial prostatitis

**DOI:** 10.3389/fphar.2023.1243824

**Published:** 2023-09-18

**Authors:** Apurva Virmani Johri, Pranav Johri, Naomi Hoyle, Lia Nadareishvili, Levan Pipia, Dea Nizharadze

**Affiliations:** ^1^ Vitalis Phage Therapy, New Delhi, India; ^2^ Eliava Phage Therapy Center, Tbilisi, Georgia; ^3^ Skagit Regional Health, Mount Vernon, WA, United States

**Keywords:** phage therapy, chronic bacterial prostatitis, *E. coli*, antibiotic resistance, biofilms, bacteriophage, successful treatment

## Abstract

**Background:** Chronic Bacterial Prostatitis (CBP) is inflammation of the prostate caused by bacterial infection. An estimated 8.2% of men have prostatitis, most commonly under the age of 50. Antibiotics often fail to treat CBP due to presence of bacterial biofilms and rising antibiotic resistance of pathogenic bacterial strains. The multidrug resistant (MDR) bacterial strains often implicated in cases of CBP include Extended Spectrum Beta Lactam resistant *Escherichia coli*, Vancomycin resistant Enterococci, Gram-positive bacterial strains like Staphylococci and Streptococci, Enterobacteriaceae like *Klebsiella* and *Proteus*, and *Pseudomonas aeruginosa*. CBP patients experience significant deterioration in quality of life, with impact on mental health comparable with patients of diabetes mellitus and chronic heart failure, leading patients to explore alternatives like phage therapy.

**Case presentation:** We present the case of a patient diagnosed with and exhibiting typical symptoms of CBP. Tests of the prostatic and seminal fluids identified *E. coli* as the causative pathogen. The patient did not experience favourable long-term treatment outcomes despite repeated antibiotic courses administered over 5 years. This led him to seek phage therapy for treatment of his condition.

**Methods and outcome:** The cultured strain of *E. coli* was tested against bacteriophage preparations developed by the Eliava Institute, Georgia. Preparations showing lytic activity against the strain were used for the patient’s treatment at the Eliava Phage Therapy Center (EPTC). The patient underwent two courses of treatment with the EPTC. The first treatment course resulted in significant symptomatic improvement, followed by complete resolution of symptoms post the second course of phage therapy. Samples tested during treatment showed declining bacterial growth, corresponding with symptomatic improvement. Post-treatment cultures had no growth of pathogenic bacteria.

**Discussion:** This case illustrates the efficacy of bacteriophages in treating CBP, a condition that is often resistant to antibiotic therapies. Antibiotics such as ofloxacin, Fosfomycin, trimethoprim, nitrofurantoin and ceftriaxone were administered in multiple courses over 5 years, but the infection recurred after each course. After two courses of phage therapy, the patient experienced long-term symptom resolution and substantial reduction in bacterial load. Increasing numbers of such cases globally warrant further research into the potential for bacteriophages for treating MDR and chronic infections.

## Introduction

Chronic bacterial prostatitis is inflammation of the prostate gland caused by a subacute, chronic bacterial infection of the prostate and associated areas of the male genitourinary system (Krieger, et al., 2008; Sharp, et al., 2010). It is diagnosed through positive bacterial cultures of samples from the infected region, which typically include expressed prostatic fluid (EPS), post-prostatic massage urine, and semen (Budia, et al., 2006; Magri, et al., 2009; Sharp, et al., 2010).

Globally, an estimated 8.2% men get prostatitis at least once in their lives, most of them under the age of 50 (Krieger, et al., 2008). The US National Institutes of Health classify prostatitis into the following categories (Krieger, et al., 1999):

Category I: Acute bacterial prostatitis (ABP).
Category II: Chronic bacterial prostatitis (CBP).
Category IIIA: Chronic prostatitis/chronic pelvic pain syndrome (CP/CPPS)—Inflammatory.
Category IIIB: CP/CPPS—Non-Inflammatory.
Category IV: Asymptomatic inflammatory prostatitis.

Symptoms of CBP can vary from patient to patient. They include, but are not limited to, urinary urgency, increased urinary frequency, burning micturition, ejaculatory pain and burning, haematospermia, pelvic and suprapubic pain, lower back pain, erectile dysfunction, and premature ejaculation (Nickel, 2012; Bowen, et al., 2015).

CBP is typically treated with long and repeated doses of antibiotics that have high lipid solubility, low protein binding and small water-soluble molecules to allow diffusion through the lipid membrane of the prostatic epithelium and reach the infected prostatic tissue (Xiong, et al., 2020). Drugs such as alpha-blockers, non-steroidal anti-inflammatory drugs (NSAID) and antipyretics may also be prescribed for symptom management (Pontari, 2002).

Antibiotic treatments can often be inadequate in controlling CBP infections in the long term (Su, et al., 2020). Recurrence of CBP is linked with the presence of bacterial biofilms commonly associated with chronic infections, an increase in antibiotic resistance in pathogenic as well as opportunistic bacterial strains, and a limited set of antibiotics that can penetrate into the prostate gland through the prostatic epithelium (Costerton, et al., 1999; Mazzoli, 2010; Sharp, et al., 2010; Wagenlehner, et al., 2014).

Recent studies have shown an evolution in the treatment options for CBP. Though fluoroquinolones have been the antibiotic of choice for the treatment of CBP, the emerging bacterial resistance amongst common uropathogens like *E. coli*, *Enterococcus faecalis* and *Staphylococcus* spp., and persistence or recurrences of infection have brought forth other, more unconventional treatments for treating CBP (Mendoza-Rodríguez, et al., 2023). This includes utilising drugs like Fosfomycin which have traditionally been used for treatment of uncomplicated urinary tract infections, as well as alternative modalities like phage therapy (Letkiewicz, et al., 2010; Su, et al., 2020; Xiong, et al., 2020; Marino, et al., 2022). Another notable result from a recent study suggests that category III prostatitis may also be caused by bacterial infection, as is CBP (Song, et al., 2023). Novel antibacterial treatment modalities may be applicable for treating such cases.

In cases of failed antibiotic treatments such as the one presented below, bacteriophage therapy (or simply, phage therapy) can be a credible alternative, and is gaining acceptability for treatment of bacterial infections, with successful treatment experiences galvanising patients and doctors around the world to explore phage therapy as a treatment option for infections that do not respond to antibiotics (Letkiewicz, et al., 2010; Górski, et al., 2018; Xiong, et al., 2020; Johri, et al., 2021; Loganathan, et al., 2021; Hatfull, et al., 2022).

For therapeutic use, purified lytic phages are tested for sensitivity against the patient’s particular pathogenic bacterial isolate. The administration for prostatic and urinary tract infections is usually systemic as well as topical, either through intravesical administration and/or rectal suppositories (Xiong, et al., 2020; Leitner, et al., 2021). Once the phages reach the site of infection, they infect their target bacteria and kill the bacterial cells through a process called lysis (Koskella and Meaden, 2013; Chanishvili, 2016; Guo, et al., 2020).

Phages are able to overcome many of the limitations of antibiotics in treating chronic bacterial infections such as CBP: They are bactericidal, as opposed to some antibiotics, which can be bacteriostatic; they are self-replicating, making them especially useful in areas of low vascularity such as the prostate gland, where drug delivery in sufficient quantities to achieve MIC can be challenging; they can penetrate through and destroy bacterial biofilms and the bacterial colonies which are often implicated in chronic and recurrent infections; they are not impacted by antibiotic resistance and have the intrinsic ability to adapt to overcome bacterial resistance to phages; their narrow host range results in minimal side effects by avoiding unintentional damage to the microbiota even with long term usage (Carlton, 1999; Loc-Carrillo and Abedon, 2011; Pires, et al., 2017; Danis-Wlodarczyk, et al., 2021; Hoyle and Kutter, 2021). Phages have been shown to have immune modulating effects and can reduce inflammation, which can potentially prevent conditions linked with chronic inflammation, such as certain cancers (Górski, et al., 2018).

The Eliava Institute of Bacteriophages, Microbiology and Virology, Georgia, has a history and experience of 100 years in developing purified therapeutic phage preparations. These preparations are prescribed and administered to patients at the Eliava Phage Therapy Center (EPTC), which offers phage therapy to local and international patients in line with the regulations of the country of Georgia. Six standard phage preparations are first tested against patients’ bacterial isolates (details in Table A1). If the isolate is not targeted by, or is resistant to, any of the standard preparations, a customised phage preparation can often be developed specifically for the patient’s particular pathogenic bacterial strain (Kutter, et al., 2010; Abedon, et al., 2011; Danis-Wlodarczyk, et al., 2021; Eliava BioPreparations, 2023).

## Case description

A 26-year-old Indian male had the following subjective symptoms from March 2013 till May 2018: penile pain and burning on ejaculation, pelvic pain post ejaculation, urinary urgency, burning micturition, fever, and general weakness and malaise in the body through the day.

Semen cultures done initially after symptoms appeared, and then again after 5 months, showed a growth of *E. coli* ≈ 1 × 10^5^ CFU/mL. A digital rectal exam (DRE) performed by a urologist revealed a boggy and tender prostate. Based on the subjective symptoms, culture reports, and DRE, the patient was diagnosed with CBP.

The patient initially had daily fever going up to 39.4°C. He was given a course of antibiotics for 1 week. After initial antibiotic therapy, he continued to have a subfebrile temperature of 37.8°C. Antipyretics (acetaminophen) were taken daily to control the fever.

Historical information from the patient indicates he was treated 14 times with the following antibiotics, for which each course lasted 4–8 weeks: oral antibiotics including fluoroquinolones—ofloxacin, levofloxacin and ciprofloxacin, Fosfomycin, trimethoprim, nitrofurantoin, and amoxicillin, as well as intravenous ceftriaxone.

After initial relief with the first course of antibiotics, the symptoms reappeared and were not fully relieved with further courses of antibiotics and would recur frequently. Due to this, the patient decided to undergo phage therapy at the EPTC.

An initial, comprehensive urological testing was conducted at the EPTC. The patient’s EPS, semen and urine samples were collected. The patient reported that the DRE and prostatic massage were painful, which the urologist correlated with a tender prostate. The samples were observed microscopically as well as cultured for aerobic bacteria. Sensitivity testing with Eliava Institute’s standard phage preparations was conducted on the bacterial strains that were cultured. Table 1 shows the outcomes of these tests.

**TABLE 1:** Results of analysis and cultures of samples from the infected region—initial testing.

Specimen name	Bacteria cultured	Growth (CFU/mL)	Eliava Institute’s standard phage preparations showing bactericidal action
Expressed prostatic secretion (EPS)	*Escherichia coli*	1 × 10^8^	Intesti bacteriophage Ses bacteriophage Enko bacteriophage
Semen	*Escherichia coli*	1 × 10^8^	Intesti bacteriophage Ses bacteriophage Enko bacteriophage
Urine	*Escherichia coli*	1 × 10^8^	Intesti bacteriophage Ses bacteriophage Enko bacteriophage

The samples did not have any fungal growth, nor was any presence of gonococcus detected in any sample. Blood tests showed normal blood counts, leukocyte counts, ESR, CRP and PSA.

*E. coli* was treated with a combination of Intesti and Ses bacteriophage preparations. For the first 10 days, a daily dose of Intesti phage was prescribed in the forms of oral liquid, rectal suppository, and intravesical administration. Concurrently, a daily dose of Ses phage was also prescribed in the forms of oral liquid and rectal suppository.

For cases of CBP, the EPTC uses the oral administration route for systemic assimilation of the phages, which can be continued on a long-term basis. The rectal route is typically used alongside the oral route because it is known to increase the phage bioavailability in the bloodstream, while the intravesical route is used to introduce the phages close to the site of infection (Xiong, et al., 2020). The rectal and intravesical routes are used for a limited duration so as not to irritate the rectal mucosal lining, or the urethral tract. Oral phages and rectal suppositories are self-administered by the patient. Intravesical administration is performed by the urologist at the EPTC.

The patient felt significant reduction in all symptoms within 7 days of starting phage therapy. His mild fever was the first symptom to resolve completely.

Two weeks after the initial testing, the patient’s EPS was tested again; results are shown in Table 2. In contrast to the first prostatic massage 2 weeks prior, the patient reported that the massage was not painful.

**TABLE 2:** Results of analysis and cultures of samples from the infected region—second round of testing.

Specimen name	Bacteria cultured	Growth (CFU/mL)	Eliava Institute’s standard phage preparations showing bactericidal action
Expressed prostatic secretion (EPS)	*Staphylococcus aureus*	5 × 10^5^	Pyo bacteriophage Intesti bacteriophage Ses bacteriophage Fersis bacteriophage Enko bacteriophage Staphylococcal bacteriophage
	*Enterococcus faecalis*	1 × 10^5^	None
	*Escherichia coli*	<1 × 10^3^	Intesti bacteriophage Enko bacteriophage

Presence of previously undetected bacterial strains was attributed to the activity of phages in breaking polymicrobial biofilms, commonly found in long standing, chronic infections such this one. *S. aureus*, *E. faecalis* and the remaining *E. coli* were treated with Intesti and Enko bacteriophage preparations, given in oral liquid and rectal suppository forms, for a total duration of 30 days. *E. faecalis* did not have sufficient *in-vitro* sensitivity to the phage preparations, and it was decided by the doctors that if the strain continued to be present in the patient’s samples in significant quantities after this treatment course, a customised phage could be prepared for it, if needed.

After this treatment course, the patient reported significant subjective improvement based on symptom relief. The only remaining symptom was mild burning on ejaculation. Nine months after this course of phage therapy had finished, he sent his EPS and semen samples to the EPTC for repeat testing. In this time, the patient did not take any antibiotics or any other antibacterial treatments. The test results are given in Table 3.

**TABLE 3:** Results of analysis and cultures of samples from the infected region—third round of testing.

Specimen name	Bacteria cultured	Growth (CFU/mL)	Eliava Institute’s standard phage preparations showing bactericidal action
Expressed prostatic secretion (EPS)	*Serratia marcescens*	1 × 10^8^	N.A. (standard phage preparations do not have a phage against this bacterium)
Semen	*Enterococcus faecalis*	1 × 10^6^	Intesti bacteriophage

Along with *E. faecalis*, the bacterium *Serratia marcescens* was cultured in this test, which was previously undetected. Since a standard phage preparation is not available against *S. marcescens*, the patient and the doctors at the EPTC together decided to initially only treat the *E. faecalis*, which showed sensitivity to Intesti bacteriophage. We considered that the given *S. marcescens* could be a normal commensal bacterium that would not likely cause infection in an immune competent host, and together with an improving clinical picture, it was not likely pathogenic in this case. However, if after this course of treatment, symptoms were to persist, and the *S. marcescens* strain was detected in repeat cultures, it would be considered pathogenic, and a customised phage would be prepared to treat the *S. marcescens*.

**FIGURE 1:**
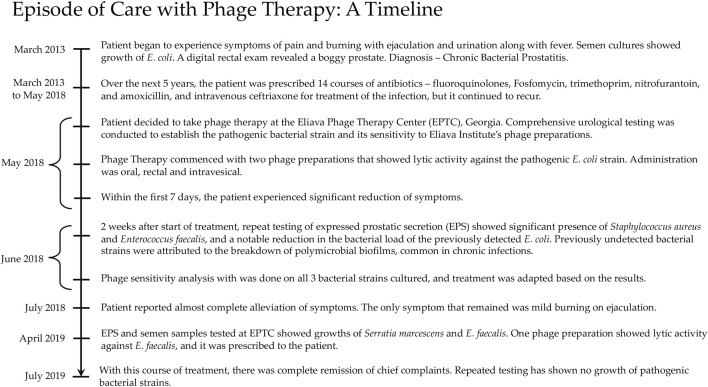
Episode of care with phage therapy: a timeline.

By the end of this course of treatment, the patient’s chief complaints were in complete remission, and have not returned since. A repeat of his semen culture has shown no growth of bacteria. A timeline from the patient’s episode of care with phage therapy is given in Figure 1.

## Discussion

Prostatitis is a notoriously difficult disease to treat causing significant morbidity and cost associated with its lengthy treatment (Pontari, et al., 2007). Sustained treatment efforts often do not provide relief to patients (Marquez-Algaba, et al., 2022). Frequent relapse of the condition and worsening of symptoms can cause significant deterioration in quality of life for CBP patients. The impairment in the mental health of sufferers is comparable with patients of diabetes mellitus and chronic heart failure (McNaughton Collins, et al., 2001; Xiong, et al., 2020).

Even with the treatment standard of 6—8 weeks of antibiotic therapy using antibiotics that have good penetration into the prostate gland, CBP is commonly associated with recurrent infections (Meyrier and Fekete, 2023). Failure of antibiotic treatment in providing long term relief is due to two main factors: the presence of bacterial biofilms, and antibiotic resistance of the bacterial pathogens (Mazzoli, 2010; Wagenlehner, et al., 2014). Biofilms are implicated in chronic infections, as they protect bacterial colonies within them from antibiotics as well as the body’s immune response, while periodically releasing planktonic bacterial cells into the host’s body (Costerton, et al., 1999; Mah & O’Toole, 2001; de la Fuente-Núñez, et al., 2013). Prostatic calcifications may be present in certain cases of CBP and can provide a favourable environment for biofilm formation (Mazzoli, 2010; Zhao, et al., 2012). It is also important to note that patients with CBP can continue to be symptomatic while no longer having a bacterial infection. This condition is known as Chronic Pelvic Pain Syndrome (CPPS), which can present as a transitional state after CBP or on its own as an initial condition. Some experts note that a trial of antibiotics is reasonable for CPPS (Anothaisintawee, et al., 2011; Pontari, 2022). We would agree that this can also hold true for phage therapy. It is possible that the mechanism has some relation to biofilm presence as described above, or as an anti-inflammatory through immune modulation (Górski, et al., 2012).

An objective indicator of infection in samples from the infected region is the presence of leukocytes in higher-than-normal concentrations (Schaeffer, et al., 1981; Wright, et al., 1994; Nickel, et al., 2006). One limitation of our study is the lack of regular microscopy throughout this patient’s testing and treatment. This could have further supported the diagnosis of CBP over CPPS (Nickel, et al., 2003).

Phage therapy is a promising treatment option for sufferers of chronic bacterial infections like CBP, for people with antibiotic allergies and those who are unable to tolerate the various side effects of antibiotics. Polyvalent phage cocktails such as the ones produced by the Eliava Institute have the advantage of preventing development of bacterial resistance, thus bolstering their robustness.

Antibiotic and antimicrobial resistance (AMR) is rising rapidly around the world and is emerging as one of the leading threats to global public health (World Health Organisation, 2020). According to a recent study, an estimated 1.27 million deaths were attributable to bacterial AMR in 2019 (Antimicrobial Resistance Collaborators, 2022). It is estimated that by the year 2050, 10 million lives will be lost each year to AMR (O’Neill, 2016). The COVID-19 pandemic has accelerated the spread of AMR due to widespread use of antibiotics (CDC, 2022; Sulayyim, et al., 2022). Additionally, during epidemics and pandemics, the presence of antibiotic-resistant bacteria in hospitals poses a serious health risk of secondary bacterial infections for hospitalised patients and compounds the complexity of managing the primary infection (Adebisi, et al., 2021; Pan American Health Organisation, 2022).

Phage therapy is a possible alternative to tackle this growing “silent pandemic” of antibiotic resistance. Antibiotic resistance does not impact phage susceptibility of a bacterial strain (Kutateladze, et al., 2016; Moghadam, et al., 2020). Moreover, phage biology indicates that lytic bacteriophages can potentially attack and clear any infection caused by extracellular bacteria (Guo, et al., 2020). In 2017, the WHO listed 12 bacterial strains as pathogens for which novel antimicrobials are needed on priority, as they have become pan-resistant to the currently available antibiotics (World Health Organisation, 2017). Bacteriophages can potentially be isolated from natural sources and purified for therapeutic use for nine of these 12 bacteria. Phages can also help contain deadly secondary bacterial infections caused by such pan-resistant superbugs during epidemics and pandemics (Manohar, et al., 2020).

As phages and antibiotics have differing and unrelated mechanisms of antibacterial action, bacterial resistance to one can result in sensitivity to the other. This phage-antibiotic synergy (PAS) can be exploited for treatment of multidrug resistant and pan drug resistant infections (Comeau, et al., 2007).

There are limitations to the utility of phage therapy given its narrow host range to its targeted bacteria, the potential for development of phage resistant bacterial strains, and the regulatory challenges in certifying a newly isolated phage. In many cases, phage therapy may only be a supplement to antibiotic therapy. While antibiotics will remain the primary treatment option for bacterial infections, especially for acute infections, phage therapy is a good alternative to antibiotics in cases of chronic, recurrent or antibiotic-resistant infections.

Currently, phage therapy is a regulated treatment option only in a few countries in the world. However, more and more countries are beginning to take the relevant steps in order to move from compassionate use as per the World Medical Association’s Declaration of Helsinki, towards regulating its use in a safe and efficacious manner on a mass level (Pirnay, et al., 2018; Voelker, 2019; Bacteriophage News, 2022).

Though phages were discovered over a hundred years ago and phage therapy has been developed and practised in certain countries since the early 21st century, the discovery of antibiotics quickly replaced phages as the mainstay of treatment of bacterial infections in most parts of the world (Carlton, 1999). However, in the face of growing antibiotic resistance, often called a silent pandemic, the potential of phage therapy needs to be widely explored as a supplement to antibiotic treatment, and to augment efforts towards antibiotic stewardship across the world.

## Conclusion

This is an example of a successful case study of treatment of a refractory case of chronic bacterial prostatitis. In this case of CBP, multiple extended treatment courses with numerous antibiotics were prescribed over a period of 5 years to treat the patient’s chronic infection. The drugs were administered both orally and intravenously but could not provide alleviation of symptoms nor eradicate clinically significant bacterial infection. In contrast, application of therapeutic phages treated the infection over two treatment courses, as exhibited by complete symptom resolution and negative cultures of samples from the infected region. The patient’s quality of life has hugely improved since the completion of his treatment with phages and has continued without relapse 4 years after finishing treatment.

Sufferers of chronic infections that cause conditions like CBP, can benefit from the development of phage therapy. Dedicated studies and rigorous trials may be required to bring about regulation for therapeutic use of phages on a large scale. Certain countries have already legislated on the research and use of phages in healthcare, paving the way for others to follow.

## Data Availability

The data analyzed in this study is subject to the following licenses/restrictions: The data presented in this study is available on request from the corresponding authors. The data is not publicly available to protect the privacy of the patient. Requests to access these datasets should be directed to AVJ, apurva@vitalisphagetherapy.com.

## APPENDIX A

**TABLE A1:** Standard Phage Preparations made by the Eliava Institute of Bacteriophages, Microbiology and Virology.

Phage preparation	Composition—Phage lysates of:	Titre (PFU/mL)
Pyo bacteriophage	*Streptococcus* spp., *Staphylococcus* spp., *Escherichia coli*, *Pseudomonas aeruginosa*, and *Proteus* spp.	≈ 10^5^
Intesti bacteriophage	*Staphylococcus* spp., *Enterococcus* spp., *Proteus* spp., *Shigella* spp., *Salmonella* spp., *Escherichia coli*, and *Pseudomonas aeruginosa*	≈ 10^5^
Fersis bacteriophage	*Staphylococcus* spp., and *Streptococcus* spp.	≈ 10^5^
Ses bacteriophage	*Staphylococcus* spp., *Streptococcus* spp., and *Escherichia coli*	≈ 10^5^
Enko bacteriophage	*Staphylococcus* spp., *Shigella* spp., *Salmonella* spp., and *Escherichia coli*	≈ 10^5^
Staphylococcal bacteriophage	*Staphylococcus* spp.	≈ 10^5^
